# Impact of Freeze-Drying on Cord Blood (CB), Serum (S), and Platelet-Rich Plasma (CB-PRP) Preparations on Growth Factor Content and In Vitro Cell Wound Healing

**DOI:** 10.3390/ijms231810701

**Published:** 2022-09-14

**Authors:** Sabrina Valente, Carmen Ciavarella, Gloria Astolfi, Elisa Bergantin, Nico Curti, Marina Buzzi, Luigi Fontana, Piera Versura

**Affiliations:** 1 DIMES, Alma Mater Studiorum University of Bologna, 40138 Bologna, Italy; sabrina.valente2@unibo.it (S.V.); carmen.ciavarella2@unibo.it (C.C.); 2 Center for Applied Biomedical Research (CRBA), Alma Mater Studiorum University of Bologna, 40138 Bologna, Italy; gloria.astolfi2@unibo.it; 3 Ophthalmology Unit, DIMES, Alma Mater Studiorum University of Bologna, 40138 Bologna, Italy; luigi.fontana6@unibo.it; 4 IRCCS Azienda Ospedaliero Universitaria di Bologna, 40138 Bologna, Italy; elisa.bergantin@aosp.bo.it (E.B.); marina.buzzi@aosp.bo.it (M.B.); 5 eDIMES Lab, DIMES, Alma Mater Studiorum University of Bologna, 40138 Bologna, Italy; nico.curti2@unibo.it

**Keywords:** freeze-drying, cord blood, serum, platelet-rich plasma, epidermal growth factor, brain-derived growth factor, wound healing, cell proliferation, cell activation, MIO-M1 cells

## Abstract

Blood-based preparations are used in clinical practice for the treatment of several eye disorders. The aim of this study is to analyze the effect of freeze-drying blood-based preparations on the levels of growth factors and wound healing behaviors in an in vitro model. Platelet-rich plasma (PRP) and serum (S) preparations from the same Cord Blood (CB) sample, prepared in both fresh frozen (FF) and freeze-dried (FD) forms (and then reconstituted), were analyzed for EGF and BDNF content (ELISA Quantikine kit). The human MIO-M1 glial cell line (Moorfield/Institute of Ophthalmology, London, UK) was incubated with FF and FD products and evaluated for cell migration with scratch-induced wounding (IncuCyte S3 Essen BioScience), proliferation with cyclin A2 and D1 gene expression, and activation with vimentin and GFAP gene expression. The FF and FD forms showed similar concentrations of EGF and BDNF in both the S and PRP preparations. The wound healing assay showed no significant difference between the FF and FD forms for both S and PRP. Additionally, cell migration, proliferation, and activation did not appear to change in the FD forms compared to the FF ones. Our study showed that reconstituted FD products maintained the growth factor concentrations and biological properties of FF products and could be used as a functional treatment option.

## 1. Introduction

Corneal epithelial turnover and healing are complex cascades of events controlled by epitheliotropic growth factors (GFs), such as epithelial growth factor (EGF) and transforming (TGF) growth factors α and β, as well as neurotrophic factors, such as nerve growth factor (NGF) and brain-derived neurotrophic factor (BDNF) [1,2]. The rationale for using blood-based eyedrops is to promote cell proliferation and migration through the supply of active substances, particularly GFs, thereby replenishing natural tears due to a lack of these substances in diseased conditions [3,4]

The topical application of blood-based eye drops was proven to be successful as a treatment option in cases of keratopathy, including severe dry eye disease (DED), corneal ulcers, persistent epithelial defects, neurotrophic keratitis (NK), ocular surface burns, ocular Graft Versus Host Disease, and limbal stem-cell deficiency [5,6]. Different preparations from both autologous (from the patients themselves) and allogeneic (from donors, either peripheral adult or cord blood) sources have been proposed, along with a preference for serum (S) or platelet-rich plasma (PRP) products [5,7].

Lyophilization, i.e., freeze-drying (FD), is a technological process in which frozen material is dried via the sublimation of ice and is widely used for plasma derivatives, vaccines, antibiotics, and biotechnological products [8,9]. These products are reduced into dehydrated powders, which can be stored at room temperature or 4 °C for long periods of time and then easily reconstituted through the addition of a solvent to re-assume the natural characteristics of the products before the FD process. The storage and delivery of FD products no longer require cold-chain logistics, resulting in considerable cost reductions and easier availability on demand.

To date, there have been no feasible solutions for dispensing blood-based eye drops other than through the liquid form stored at a temperature of −20 or −80 °C, depending on the storage length. The following evidence on the efficacy of FD products in the treatment of orthopedic and dental diseases [10,11], FD-autologous serum (AS) [12], and FD-plasma rich in growth factors (PRGF) [13] eyedrops have also been explored.

The purpose of this work is to evaluate the effect of freeze-drying on products from cord blood (CB) as a source prepared as both serum (S) and platelet-rich plasma (PRP). EGF and BDNF levels were chosen to analyze the effect of FD, as they are the most represented epitheliotrophic and neurotrophic factors, respectively, in blood products [14]. The analysis was accomplished by comparing the same S or PRP samples, manufactured as either fresh-frozen (FF) or FD preparations. The biological effect on cell migration, proliferation, and activation exerted by these differently obtained products was then evaluated with an in vitro wound healing assay using a cell line responding to GF supplementation.

## 2. Results

The demographic characteristics of the CB sample donors (i.e., mothers and babies) are reported in Table 1. No differences were found between the two preparations in terms of the population included.

### 2.1. Growth Factor Levels

The levels of BDNF and EGF were recorded in all the pools analyzed. The levels of EGF and BDNF in the two preparations (S and PRP) and the two procedures (Freeze-Dried (FD) and Fresh-Frozen (FF)) are summarized in Table 2. A statistically significant difference between FF and FD samples was found only for BDNF in both the S and PRP preparations.

In Table 3, we report the analysis performed considering the FF and FD variables as paired. In this case, we did not observe any statistical difference between the PRP and S samples for the BDNF and EGF growth factors.

**Figure 1:**
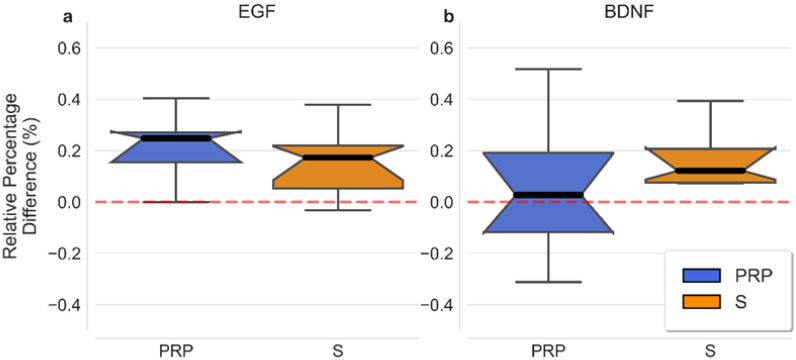
Distributions of the relative percentage values (i.e., the difference between FF and FD normalized by FF for the two preparations (PRP and S) in the two growth factors ((**a**) EGF and (**b**) BDNF). PRP and S distributions are represented separately for comparison. Each distribution is represented independently. Minimum and maximum distributions are shown by lines and the median using an internal notch (representing the uncertainty of the median). The box spread represents the 1st and 3rd quartiles of distribution. EGF: Epithelial Growth Factor; BDNF: Brain-Derived Neurotrophic Factor; S: Serum; PRP: Platelet-Rich Plasma.

A comparison of the different preparations with respect to the levels of GFs is shown in Figure 1, which illustrates the differences between the fresh-frozen and freeze-dried concentrations, normalized according to the fresh-frozen samples (i.e., the relative percentage).

### 2.2. Cell Model

**Figure 2:**
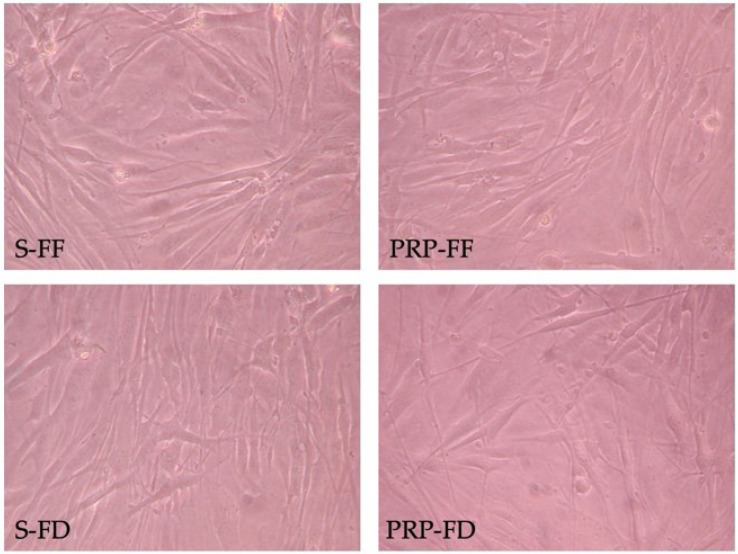
MIO-M1 cell culture. Representative images of MIO-M1 cells cultured in a DMEM culture medium supplemented with 5% Fresh Frozen serum (S-FF), Freeze-dried (S-FD) serum, Fresh Frozen Plasma-Rich Platelet (PRP-FF), or Freeze-dried PRP (PRP-FD).

The MIO-M1 cells grown with the two products added to the culture medium exhibited a spindle-like morphology with long cytoplasmic projections and tubular processes extending to adjacent cells, as well as an irregular membrane and bipolar morphology. After 48 h, the MIO-M1 cells formed a cell monolayer, and no significant morphological differences were observed between the products (Figure 2).

#### Scratch Wound Assay

**Figure 3:**
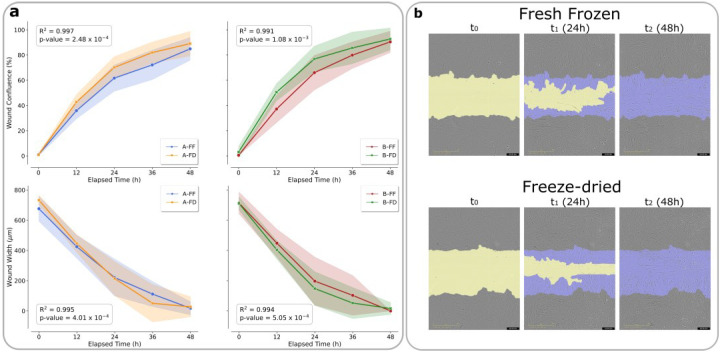
Analysis of the scratch wound on MIO-M1 treated with S-FF and S-FD. (**a**) Graphical representation of the wound confluence and wound width of MIO-M1 cells treated with 5% S-FF and S-FD preparations added to the growth medium; A = sample A, and B = sample B. (**b**) Representative images of MIO-M1 cells recorded using the IncuCyte S3 instrument with a 10x objective lens at different treatment times with S-FF and S-FD (t_0_ = at the scratch, left column; t_1_ = 24 h, middle column; t_2_ = 48 h, right column). Yellow: initial scratch wound area; purple: MIO-M1 cells that migrated into the wound area.

**Figure 4:**
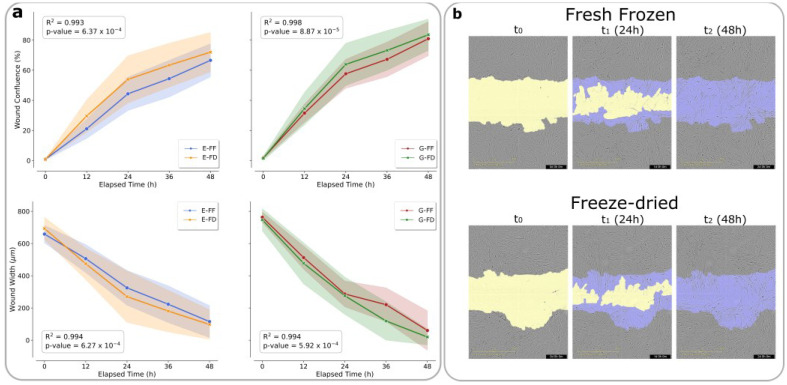
Analysis of the scratch wound on MIO-M1 treated with PRP-FF and PRP-FD (**a**) Graphical representation of the wound confluence and wound width of MIO-M1 cells treated with 5% PRP-FF and PRP-FD preparations added to the growth medium; E = sample E, and G = sample G. (**b**) Representative images of MIO-M1 cells recorded using the IncuCyte S3 instrument with a 10x objective lens at different treatment times with PRP-FF and PRP-FD (t_0_ = at the scratch, left column; t_1_ = 24 h, middle column; t_2_ = 48 h, right column). Yellow: initial scratch wound area; purple: MIO-M1 cells that migrated into the wound area.

The effects of S and PRP products on the MIO-M1 cell wound healing capacity were assessed through a scratch wound assay. MIO-M1 cells were able to progressively occupy the initial wound, closing it completely at 48 h post-wounding, with exposure to S-FF, S-FD, PRP-FF, and PRP-FD derived from specific samples. Data were coherent with the reduction in wound width (Figure 3a,b and Figure 4a,b). No microbial growth was observed after the reconstitution of FD products.

### 2.3. Real-Time PCR for Proliferation Genes

**Figure 5:**
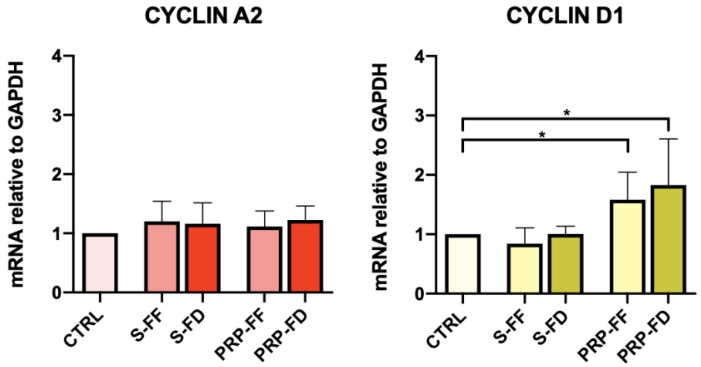
Cyclin A2 and Cyclin D1 mRNA levels in MIO-M1 cells post-scratch assay at 48 h. Gene expression analysis of the proliferation markers Cyclin A2 and Cyclin D1 was performed using Real-Time PCR. Results are reported as the fold changes relative to untreated MIO-M1 cells cultured in DMEM L-Glutamax plus 5% FBS and represented as the mean ± standard deviation. Experiments were performed in triplicate. Statistical analyses were performed using one-way ANOVA followed by Tukey’s multiple comparisons test. *, *p* < 0.05.

The cyclin A2 and D1 genes were analyzed to evaluate cell proliferation following treatment with S and PRP. We did not observe significant differences between the FF and FD preparations for both. In particular, cyclin A2, which is specific for DNA replication during the S phase of the cell cycle, was similar under all treatment conditions. Cyclin D1, which promotes G1/S transition, was up-regulated in the PRP-FF and PRP-FD preparations compared to the control cells, but no significant differences between PRP-FF and PRP-FD were detected (Figure 5).

### 2.4. Real-Time PCR for Migration and Activation Genes

**Figure 6:**
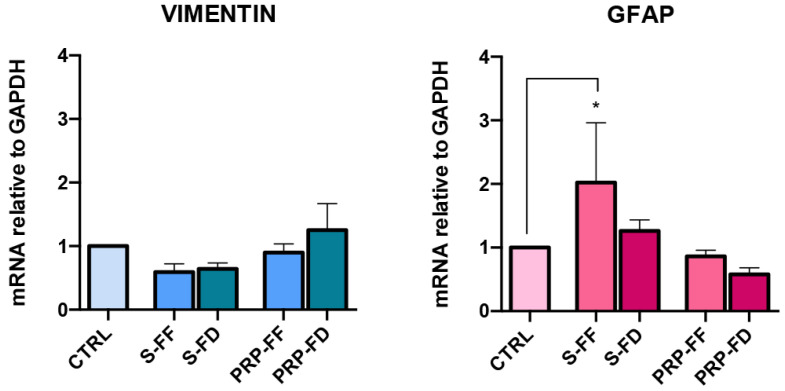
VIMENTIN and GFAP mRNA levels in MIO-M1 cells post-scratch assay at 48 h. Gene expression analysis of the migration marker Vimentin and the activation marker GFAP was performed using Real-Time PCR. Results are reported as the fold changes relative to untreated MIO-M1 cells cultured in DMEM plus 5% FBS and represented as the mean ± standard deviation. Experiments were performed in triplicate. Statistical analyses were performed using one-way ANOVA followed by Tukey’s multiple comparisons test. GFAP: * *p* = 0.0308 CTRL vs. S-FF.

The gene expression of Vimentin was analyzed to investigate the migratory potential in MIO-M1 after the scratch assay. As shown in Figure 6, in both FF and FD preparations, Vimentin mRNA did not display significant changes under treatment with S and PRP. The gene expression of GFAP, a marker associated with retinal glial activation after damage, was also investigated. GFAP mRNA levels were found to be statistically increased in S-FF compared to those in the control cells. A non-significant decrease was observed in S-FD when compared to S-FF. Expression levels similar to those under control conditions were observed in both PRP preparations. (Figure 6).

## 3. Discussion

Data from the present study show that products from cord blood are not impaired by the freeze-drying and reconstitution process when compared to fresh products, in terms of their levels of EGF and BDNF, their wound healing capacity, and the migration and proliferation behaviors of glial Muller cells.

This study confirms that lyophilized S and PRP powder are stable at room temperature for up to 30 days and remain uncontaminated. These results agree with a previous study on peripheral blood (PB), where the time of storage was extended up to 3 months [15]. The present study focused on preparations from cord blood, a source showing significantly higher levels of GF supply compared to PB [16,17]. The S preparations presented higher GF levels than PRP, which disagrees with a previous study [14] where determinations were performed using a multiplex approach. However, in the present work, an ELISA technique was used. This highlights the well-known issue of a lack of standardization of analytical methods, as demonstrated in other papers, which suggest caution when comparing the results obtained by different methods, including multiplex versus ELISA determinations [18,19,20]. In addition, the large fluctuations that biological data are subjected to did not allow us to statistically prove the absence of biases in the two preparations. However, integrated analyses confirmed that lyophilized products maintained the same levels of EGF and BDNF observed in the corresponding fresh samples.

The impact of the lyophilization procedure on peripheral adult-blood-derived eyedrops in the treatment of ocular surface diseases was previously evaluated with in vitro models utilizing corneal cells in both [15] animals [13] and humans [12]. These studies evaluated corneal wound healing and showed no difference compared to the not lyophilized products. The novelty of the present work was to analyze the impact of lyophilization on cord blood preparations. The data confirmed that this procedure did not affect the final product compared to the non-lyophilized one.

An emerging field of interest lies in the application of blood-based products as neuroprotective agents in neurodegenerative diseases [21,22]. In particular, the focus has been given to the role of glial Muller cells in retinal neuroprotective pathways [23] in terms of the protective effects of the growth factor supply [24]. In this pathway, growth factors might also be provided by blood-based products via delivery systems, beyond the injection procedures, that are actively being explored [25,26]. To further explore this issue, the present study used a human cell line of retinal glial cells, MIO-M1 cells, which were recognized to be sensitive to GF supplementation.

Previous research showed the efficacy of cord blood serum (CB-S) at ameliorating the effects of oxidative stress and inflammation on the viability of rat and human Muller cells [27] by modulating the state of activation which typically occurs when glial cells are exposed to tissue damage. In the present study, the effect of lyophilized products from CB as a source was explored in MIO-M1 cells in terms of wound healing.

The analysis of cell migration was performed automatically using a scratch assay with a protocol and equipment for real-time monitoring of cellular motility, providing homogeneous scratch and image-based dynamic high-throughput analysis on wound width reduction and corresponding increases in wound confluence.

Data were consistent in showing complete MIO-M1 wound closure at 48 h post-scratch in both the FF and the FD forms and both the S and PRP preparations. The effect of wound healing was not associated with cell proliferation since the expression of the cell cycle regulators cyclin A and cyclin D1 did not undergo significant variations. Only a slight increase in cyclin D1 (not statistically significant) was observed in PRP-treated cells, potentially in accordance with the effects of this protein on cell migration. Indeed, apart from its role in regulating cell proliferation and the G1/S transition, cyclin D1 promotes cell migration via Rho-activated kinase (ROCK) and Thrombospondin-1 (TSP-1), proteins crucially involved in cell adhesion and the suppression of cell motility [28].

The mRNA expression levels of vimentin were analyzed (considering its role during cell migration), and no significant changes were observed when comparing the FF and FD products. To exclude a possible induction of MIO-M1 activation under the wound healing assay, we also performed an analysis of GFAP expression. Indeed, GFAP is a marker of glial activation under retinal damage and was found to be increased in retinal diseases [29]. As in the present analysis, no significant changes were observed when comparing the FF and FD products.

Freeze-drying offers several advantages, including (i) a long shelf life, even at room temperature, if adequately packaged; (ii) protection from microorganisms; (iii) ease of transportation and storage of the product; and (iv) easy reconstitution of the product at scalable concentrations to dispense the product with calibrated dosages based on clinical parameters or frequency of administration. Although preservation of the characteristics of the starting product was observed in the present study, the freezing injury could induce protein denaturation [30]. This result suggests that further studies are required to validate a cryoprotective agent during the process that does not interfere with biological activity after reconstitution.

## 4. Materials and Methods

The study was performed following the principles of the Declaration of Helsinki, approved by the Ethical Committee of the Policlinico S. Orsola Malpighi (100/2016/O/Sper).

In total, 230 blood samples not suitable for storage and transplantation were obtained from the Emilia Romagna Cord Blood Bank and utilized for this study.

### 4.1. Cord Blood (CB) Collection

All steps from recruitment to processing and registration of CB were performed according to the guidelines edited by the Foundation for the Accreditation of Cellular Therapy (FACT) and the Italian regulation. The CB collection for transplantation purposes was performed when the placenta was still in utero by puncturing the umbilical vein with a sterile system (Cord blood collection set, JMS, Singapore) in a bag containing 20 mL citrate-phosphate-dextrose (CPD). Serum was collected from ex utero placenta vessels in 9 mL vacuum tubes (Biomed Device, Modena, Italy) without any anticoagulant. For subsequent procedures, CB units were sent to the Processing Facility (PF) laboratory, where they were processed within 48 h from the collection.

#### 4.1.1. Assessment of CB Units

Cord blood was collected from spontaneous term births free of complications and Caesarean births (≥37th week of pregnancy), as determined by trained and qualified health personnel. The units underwent a series of checks and tests to establish the blood’s characteristics and suitability for preservation and therapeutic use. Maternal infectious disease marker (HIV, HCV, HBV Treponema pallidum, CMV, Toxoplasmosis, and HTLVI/II) evaluations were also performed. The number of white blood cells (WBCs), total nucleated cells (TNCs), and platelets (PLTs) were counted with an auto-analyzer (XN-1000, Sysmex) and expressed as the number × 10^6^ × mL. The AB0 blood group was also measured (Neo, Immucor Gamma).

#### 4.1.2. Obstetric Data

Data were retrieved anonymously from clinical records with respect to the gestational age of the mother, parity, and sex of the child.

### 4.2. Serum Preparation

Cord blood collected without an anticoagulant was allowed to clot for 24–36 h at 4 °C due to the temporary storage used before transportation from the delivery room to the PF, which was often located in another city. S was obtained via centrifugation of the blood at 3500× *g* for 10 min to remove clots. Then, the supernatant was carefully removed, aliquoted, and stored in a freezer at −80 °C.

### 4.3. PRP Preparation

To obtain PRP, the whole CB was centrifuged using low-speed centrifugation at 200× *g* for 10 min and transferred into a transfer bag for a second round of centrifugation at 2500× *g* for 10 min; then, the CB was diluted to obtain a PLT concentration between 700 and 900/microliter. The obtained PRPs were stored in a freezer at −80 °C.

For the best chance at making natural biological variability more uniform, 6 to 12 sera or PRPs were pooled based on ABO blood groups, as requested by the current legislation. In particular, 133 CB-S samples were used to produce 15 pools, and 132 CB- PRP samples were used to produce 18 pools, with both pools being approximately 4 mL each.

S and PRP with high and low values of EGF and BDNF were included in each pool in order to have more similar and comparable samples.

Each pool was divided into two parts: one part was stored at −20 °C until further use (this product was designated as Fresh-Frozen (FF)), while the other was subjected to a lyophilization cycle, as described below. The sterility of each pool was tested before running the cycle.

### 4.4. Freeze-Drying and Reconstitution Process

S and PRP lyophilization was performed according to a lyophilization cycle with 1% water residue at the Istituto Ortopedico Rizzoli in Bologna, Italy. Samples were processed using a Genesis 25 EL Pilot Lyophilizer (SP VirTis, SP Industries, Warminster, PA, USA) under sterile conditions. The lyophilization cycle was divided into a freezing phase at −65 °C for 3 h and a lyophilization phase consisting of two subsequent steps: primary drying at −50 °C for 24 h at a pressure of 75 mT and secondary drying at 20 °C for 12 h at a pressure of 75 mT. This product was named Freeze-Dried (FD). The lyophilized CB serum and PRP were stored at room temperature in silica gel for up to 30 days; then, the samples were reconstituted in sterile water with the same volume as the original and stored at −20 °C for up to 30 days until GF determination and use in the cell culture, at which point a sterility check was also performed.

### 4.5. Growth Factor Dosage

Samples from the two preparations were tested for the presence of BDNF (Brain-Derived Neurotrophic Factor) and EGF (Epidermal Growth Factor).

Samples were thawed before each assay and evaluated using a commercially available ELISA Kit (Quantikine ELISA, R&D System, Inc, Minneapolis, MN, USA) following the manufacturer’s instructions. Ranges of detectable BDNF levels were 15.6–1.000 pg/mL. Ranges of detectable EGF levels were 3.9–250 pg/mL. A Multiskan Sky microplate spectrophotometer (Thermofisher, Waltham, MA, USA) was used for analysis.

### 4.6. Cell Model and Culture Conditions

The spontaneously immortalized human Muller cell line MIO-M1 (Moorfields/ Institute of Ophthalmology-Muller 1) was obtained from the UCL Institute of Ophthalmology, London, UK20, and used as a cell model for testing the biological activity of S and PRP in the FF and FD preparations.

In accordance with the manufacturer’s instructions, MIO-M1 cells were cultured in a DMEM L-Glutamax medium (Gibco, Thermofisher Scientific, Waltham, MA, USA) plus 10% Fetal Bovine Serum (FBS) in an incubator at 37 °C with 5% CO_2_ and used to perform in vitro experiments.

#### 4.6.1. Scratch Wound Assay

The migratory ability of MIO-M1 cells treated with the four preparations (FF-S, FD-S, FF-PRP, and FD-PRP) was investigated through a scratch wound assay. For this assay, 10^4^ MIO-M1 cells/well were plated in a 96-well Essen ImageLock plate (Essen Bioscience, Ann Harbor, MI, USA) and cultured in DMEM L-Glutamax (Gibco, Thermofisher Scientific, Waltham, MA, USA) with 5% FBS near 100% confluence. Then, the cell monolayer was scratched using a WoundMaker (Essen BioScience, Ann Harbor, MI, USA) device to simultaneously create wounds in each well, washed in PBS, and treated with 100 µL DMEM L-Glutamax medium containing 5% S-FF, S-FD, PRP-FF, and PRP-FD preparations at 37 °C in an incubator with 5% CO_2_.

The culture plate was placed in an IncuCyte S3 instrument (Essen BioScience, Ann Harbor, MI, USA) and kept in a dedicated incubator. Then, each wound image per well was automatically recorded with a 10X objective lens every hour for 48 h using the IncuCyte S3/SX1 optical module phase contrast. Images were processed by using the IncuCyte 2019B software to analyze, over time, two integrated measures characterizing the movement behavior. Specifically, the wound confluence was estimated as the wound region area occupied by cells and expressed as a percentage (%), and the wound width was estimated as the distance between wound edges and expressed in micrometers (μm).

#### 4.6.2. Gene Expression Analysis

At the end of the scratch wound assay, MIO-M1 cells treated with the two preparations (S and PRP in both FF and FD) were processed for the gene expression analysis of Cyclin A2, Cyclin D1, VIMENTIN, and GFAP. The total RNA from each sample was extracted using a TRIzol Reagent (Life Technologies, Carlsbad, CA, USA) following the manufacturer’s instructions. cDNA synthesis was performed with an iScriptTM cDNA synthesis kit (BioRad Laboratories) in accordance with the manufacturer’s datasheet. Real-Time PCR analysis was carried out on a CFX Connect Real-Time PCR Detection System (BioRad Laboratories, Hercules, CA, USA) and QuantStudio™ 5 Real-Time PCR System (Applied Biosystems^®^ Laboratories, Hercules, CA, USA)/CFX-96TM Real-Time Detection System (BioRad Laboratories) using the SYBR green (Sso AdvancedTM Universal Sybr Green Supermix; BioRad Laboratories Hercules, CA, USA) and TaqMan (TaqMan Master Mix, Life Technologies, Carlsbad, CA, USA) approaches. Primer pairs (purchased from Merck Life Science, Darmstadt, Germany and IDT, Newark, NJ, USA) and probes (Life Technologies, Carlsbad, CA, USA) employed target gene expression normalized with glyceraldehyde 3-phosphate dehydrogenase (GAPDH). The relative quantification of mRNA expression was calculated with the 2ΔΔCt method and expressed as fold changes relative to the untreated control cultured with the growth medium plus 5% FBS.

The primer gene sequences used for evaluating cell proliferation and migration were as follows: GAPDH: Fwd: AATGGGCAGCCGTTAGGAAA and Rev: AGGAGAAATCGGGCCAGCTA; CYCLIN A2: Fwd CGGTACTGAAGTCCGGGAAC and Rev GGTGCAACCCGTCTCGTCTT; CYCLIN D1: Fwd ACAGATCATCCGCAAACACG and Rev TCTGGAGAGGAAGCGTGTGA; and VIMENTIN: Fwd ATCGATGTGGATGTTTCCAA and Rev TTGTACCATTCTTCTGCCTC. Probes for the TaqMan methods were Human GFAP Hs00909233_m1 (used to evaluate cell activation) and Human GAPDH Hs02786624_g1 (the housekeeping gene).

### 4.7. Statistical Analysis

Statistical analyses were performed using the statsmodels (v0.13.2) package in Python (v3.9) [31]. Descriptive statistics for the tests and variables analyzed in the subjects were reported as the geometric mean with a 95% confidence interval. The difference between the two preparation types (fresh-frozen and fresh-dried) was estimated using a two-sided *t*-test (on the geometric means), and the *p*-values were adjusted for multiple testing using the Benjamini–Hochberg method, with 0.05 considered the threshold for significance. The GraphPad Prism software v6 was used to perform statistical analyses and create graphs related to gene expression. Differences between the two preparations (S and PRP in both FF and FD) were assessed using a one-way ANOVA followed by Tukey’s multiple comparisons test. Each experiment was executed at least in triplicate. Data were expressed as the mean ± standard deviation (SD), and *p* < 0.05 was considered statistically significant.

## 5. Conclusions

The present study showed that lyophilized cord blood-S and -PRP maintained EGF and BDNF levels compared to the fresh product and were stable for up to 1 month at RT, without the use of any cryoprotectant. Lyophilized S and PRP also preserved their biological activities in a glial Muller cell in vitro model, avoiding activation or proliferation.

## Figures and Tables

**Table 1:** Summary of the characteristics of mothers and babies, donors of the CB samples.

Characteristics	PRP	S
Samples (n)	132	133
Mother’s age * (years)	32 (21–42)	33 (18–45)
Gestational age * (weeks)	40 (39–42)	40 (39–42)
Parity = 1	12	11
Parity > 1	20	22
ABO blood group	0 = 62	0 = 54
	A = 60	A = 42
	B = 8	B = 25
	AB = 2	AB = 12
Baby’s sex	61 M; 71 F	64 M; 69 F

* Data are expressed as the median (95% confidence interval).

**Table 2:** Summary of the statistics extracted from the dataset. Each concentration was converted into a logarithmic (base 10) scale to standardize the concentration. We evaluated the geometric mean and 95% confidence intervals (mean ± std) of each concentration for each preparation in both preparation types (fresh-frozen and fresh-dried). We then compared the fresh-frozen and fresh-dried groups using a two-sided *t*-test to compute the associated *p*-value. These *p*-values were adjusted (*q*-values) using the Benjamini–Hochberg correction for multiple tests. We used one or multiple stars (*) for the significance level of the test (0.001, 0.01, 0.05).

GFs	Product	Fresh-Frozen (95% CI)	Freeze-Dried (95% CI)	*q*-Value
EGF pg/mL	S	1299.12 (717–2355.44)	1193.69 (451–3162.19)	0.38
	PRP	852.79 (249–2924.18)	794.7 (267–2363.79)	0.38
BDNF pg/mL	S	22,576.52 (17,009–29,966.16)	18,903.22 (12,885–27,732.64)	0.0057 **
	PRP	16,602 (7505–36,730.21)	12,848.15 (5276–31,287.37)	0.0028 **

EGF: Epithelial Growth Factor; BDNF: Brain-Derived Neurotrophic Factor; S: Serum; PRP: Platelet-Rich Plasma.

**Table 3:** Summary of the statistics extracted from the dataset. Each concentration was converted into a logarithmic (base 10) scale to standardize the concentration. We evaluated the geometric mean and 95% confidence intervals (mean ± std) of each concentration for each preparation, considering the two preparation types (fresh-frozen and fresh-dried) as paired variables. We compared the PRP and S groups using a two-sided *t*-test computing the associated *p*-value. These *p*-values were adjusted (*q*-values) using the Benjamini–Hochberg correction for multiple tests.

GFs	PRP (95% CI)	S (95% CI)	*q*-Value
EGF pg/mL	1.07 (1–1.76)	1.09 (1–2.19)	0.9
BDNF pg/mL	1.29 (1–1.73)	1.19 (1–1.61)	0.5

EGF: Epithelial Growth Factor; BDNF: Brain-Derived Neurotrophic Factor; S: Serum; PRP: Platelet-Rich Plasma.

## Data Availability

The data presented in this study are available on motivated request from the corresponding author.
